# Schistosomiasis, endless endemicity since the ancient Egyptians

**DOI:** 10.1186/s12879-025-12356-6

**Published:** 2026-01-10

**Authors:** Ahmed Ramadan, F. Sabry, Ahmed M. Gaheen, Amr EL Rabat, El-Malky Mohamed, Abdelaleem Helal, M. Eissa, Shaimaa A. Farag, Gamal A. Badra, Marwa El fayoumy

**Affiliations:** 1 Hepatology and Gastroenterology Unit, Internal Medicine Department, Faculty of Medicine, Mansoura University, Mansoura City, Egypt; 2 Parasitology Department Faculty of Medicine, Mansoura University, Mansoura City, Egypt; 3 Hepatology and Gastroenterology Department, National Liver Institute, Menoufia University, Shebeen EL-Kom City, Egypt; 4 Clinical and Molecular Parasitology Department, National Liver Institute, Menoufia University, Shebeen EL-Kom City, Egypt

**Keywords:** Schistosomiasis, Colonoscopy, Prevalence, Crush biopsy (squash) test

## Abstract

**Background/aims:**

Human schistosomiasis is a significant and, regrettably, overlooked tropical illness. This study was to examine the frequency and features of colonic schistosomiasis in patients exhibiting colonic symptoms at the endoscopy units of Mansoura University and the National Liver Institute (Menoufia University), Egypt.

**Patients and methods:**

This study included 130 patients who were referred for colonoscopy due to various symptoms. They underwent comprehensive history taking, abdominal ultrasonography, and ileocolonoscopy with biopsies for a crush biopsy test after providing a written informed consent.

**Results:**

Sixteen (12.3%) patients were confirmed to have schistosomiasis. Bleeding per rectum, followed by chronic abdominal pain, were the predominant complaints of Schistosoma infection among the cases. Ultrasound findings showed that 25% of positive cases had organomegaly (18.8% hepatosplenomegaly and 6.3% splenomegaly). Mucosal erythema and inflammation (n = 10, 62.5%) were the most often observed morphologic features during colonoscopy in positive patients. Polyps (n = 6, 37.5%) and mucosal ulcerations (n = 6, 37.5%) were the next most common findings, either alone or in combination.

**Conclusion:**

Colonic schistosomiasis remains endemic in the Egyptian Nile Delta, with a frequency of 12.3% among the study’s patient population. The crush biopsy test is a simple and reliable technique for detecting both recent and chronic colonic schistosomiasis infections and could be employed to screen patients undergoing colonoscopy.

**Clinical trial number:**

Not applicable.

## Introduction

Schistosomiasis is a parasitic disease caused by blood flukes (Trematodes) of the genus *Schistosoma* (*S*). In 2016, the World Health Organization (WHO) reclassified schistosomiasis as a neglected tropical disease (NTD). In Egypt, evidence of schistosome eggs and antigens has been found in ancient mummies dating back to the Pharaonic era (3200 B.C.). *Schistosoma haematobium* and *S. mansoni* are known to cause serious illnesses in humans in Egypt, particularly in rural areas, due to their high prevalence and disease morbidity [1].

Globally, around 250 million individuals are estimated to be infected, with transmission occurring in 77 tropical and subtropical countries. By the late 20th century, schistosomiasis became endemic throughout the Middle East and North Africa (MENA) region [2, 3]. Among parasitic diseases, schistosomiasis ranks second only to malaria in both infection burden and population at risk [4].

In Egypt, *S. mansoni* is the dominant species, leading primarily to intestinal schistosomiasis and hepatobiliary complications. Clinical manifestations include diarrhea, rectal bleeding and abdominal pain, reflecting granuloma formation and chronic intestinal inflammation. *Schistosoma* infection can lead to long-term effects such as malnutrition, impaired growth in children, and serious liver conditions, including ascites, esophageal varices, periportal fibrosis, and eventually liver cirrhosis [5].

Following the discovery of the parasite’s life cycle in 1915, Egypt launched control programs emphasizing snail eradication [6]. Subsequent national campaigns and mass chemotherapy significantly reduced disease prevalence from approximately 3% in 2003 to 0.3% in 2012 [7]. However, ongoing transmission is driven by widespread snail hosts, poor sanitation, limited health education, and lack of treatment options especially among high-risk groups like farmers and fishermen [8].

The gold standard technique for diagnosing schistosomiasis is the microscopic detection of *Schistosoma* eggs in the stool or urine samples of suspected patients. However, the use of eggs as a screening tool may miss the identification of an active infection and potentially dangerous complications due to intermittent egg shedding. Additionally, serological and antigen-based tests in blood and urine are acceptable; however, the results of these tests cannot distinguish current from past infection [5].

Rectal biopsy examination offers higher sensitivity and is particularly valuable for confirming residual or recurrent infections in patients with negative serology. Colonoscopic evaluation is crucial in intestinal schistosomiasis because it allows direct visualization of characteristic lesions, while mucosal biopsies provide definitive histopathological confirmation [5].

Therefore, the objectives of the current study were to determine the frequency of colonic schistosomiasis through rectal biopsy examination by crush (squash) technique and to investigate the features of the disease in patients indicated for colonoscopy who visited the endoscopy units of the Departments of Hepatology and Gastroenterology at Mansoura University and the National Liver Institute Hospital, Menoufia University, Egypt.

## Patients and methods

### Ethical consideration

The Medical Research Ethical Committee, Institutional Review Board (IRB), Mansoura Faculty of Medicine, Mansoura University, approved this study, assigned ethical code number R.24.03.2535. All participants in this study supplied written informed consent, and all procedures were thoroughly described to them.

### The sample size calculation

was predicated on the prevalence of schistosomiasis in Egypt, utilizing the most similar research [9]. Utilizing Epi Info version 7.2.4.0 to determine sample size based on an anticipated prevalence of 6% with a 95% confidence interval and an acceptable margin of error of 5%, the sample size must be no less than 87 cases.

### Study design

This cross-sectional study involved 130 symptomatic patients admitted for colonoscopy at the endoscopy units of the Department of Hepatology and Gastroenterology at Mansoura University and the National Liver Institute Hospital at Menoufia University, Egypt. Inpatients and outpatients were enrolled twice weekly. Participants were inhabitants of the Dakahlia and Menoufia governorates, situated in the northeastern and southern areas of the Nile Delta. Data gatherings occurred during a six-month duration, from June to December 2024.

### Inclusion criteria

Eligible participants were adults (≥ 18 years) of either sex who presented with colonic manifestations such as rectal bleeding, altered bowel habits, abdominal pain, or positive fecal occult blood test results.

### Exclusion criteria

The study excluded pregnant women, individuals under 18 years of age, and those unwilling or unable to provide informed consent.

### Clinical and laboratory assessment

Every participant underwent a comprehensive medical history and clinical assessment. Demographic data, encompassing age and residential area, was documented alongside presenting symptoms. Standard laboratory assessments comprised a complete blood count (CBC) conducted with the Beckman Coulter LH 750 Hematology Analyzer [5].

### Abdominal ultrasonography

Ultrasound examinations were performed on the day of colonoscopy. To minimize interference from bowel gas, all subjects were examined in a fasting state using grayscale ultrasonography [10].

### Colonoscopy examination

Ileocolonoscopy was performed for all patients following a one-day bowel preparation using four sachets of polyethylene glycol electrolyte solution (MOVIPREP, Norgine Limited, UK, or an equivalent local product). Patients consumed one liter of solution (two sachets) the evening before and another liter on the morning of the procedure. Most colonoscopies were performed under conscious sedation using midazolam and opioids. Mucosal biopsies were taken from the entire colon segments as well as the morphologically detected lesions. Endoscopic abnormalities included mucosal erythema and congestion, friability, ulceration, loss of vascular pattern, granular or irregular surfaces, exudate formation, and polypoid lesions [10, 11].

### Crush (Squash) biopsy technique

Since adult worms migrate retrogradely to the pelvic venous plexus for egg deposition, the rectosigmoid region was targeted. Three tissue fragments were obtained from the anorectal fold approximately 8 cm above the anal verge using sigmoidoscopic biopsy forceps. Samples were compressed between two glass slides and microscopically examined for *Schistosoma* ova [10].

### Statistical analysis and data interpretation

Statistical analysis was conducted using SPSS software (version 26; SPSS Inc., Chicago, IL). Qualitative variables were expressed as frequencies and percentages, while quantitative variables with normal distribution confirmed via the Kolmogorov-Smirnov test were reported as mean ± standard deviation (SD). A p-value below 0.05 was considered statistically significant. Chi-square and Fisher’s exact tests were used to compare qualitative data between the groups.

## Results

Using the crush biopsy method, colonic schistosomiasis was confirmed in 16 of 130 evaluated patients (12.3%) (Tables 1 and 2). The highest infection rate was recorded among individuals aged 30–60 years (68.8% of positive cases). A rural predominance was observed (56.3% rural vs. 43.8% urban), and infection was markedly higher among males (81.3%). Rectal bleeding (31.3%) was the most frequent presenting symptom, followed by abdominal pain (25%). Ultrasonographic assessment revealed organomegaly in 25% of infected patients (hepatosplenomegaly in 18.8% and splenomegaly in 6.3%).

**Fig. 1:**
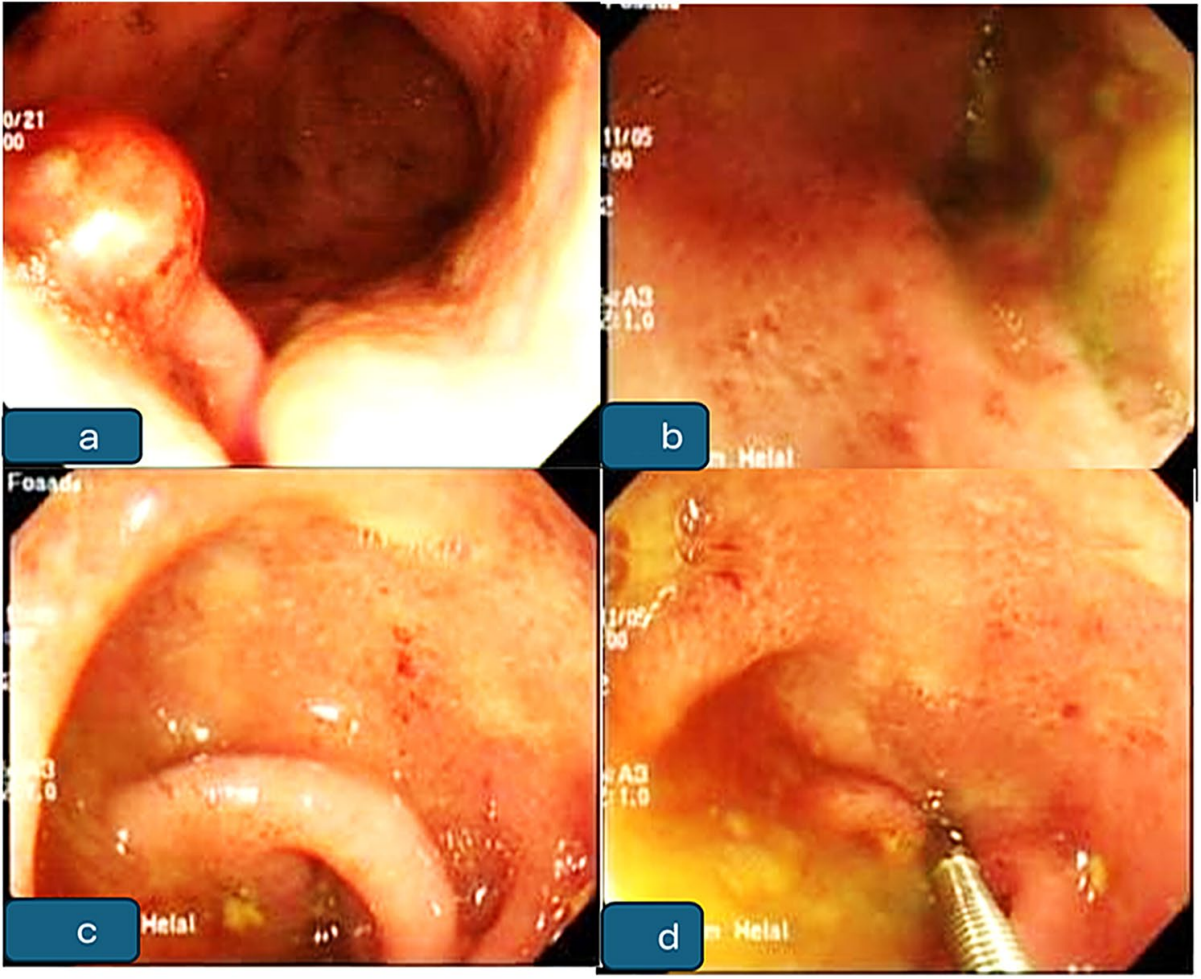
Endoscopic findings suggestive of schistosomal colonic disease: (**a**) Large polyp in sigmoid colon in chronic colitis; (**b**, **c**, **d**) Sigmoid hyperemia with excess mucus with colitis

During colonoscopy, the most frequently observed morphologic features in positive patients were mucosal erythema and congestion (*n* = 10, 62.5%), polyps (*n* = 6, 37.5%) with a size of 0.5–1 cm and it was limited to the rectum and sigmoid regions, and mucosal ulcerations (*n* = 6, 37.5%). These findings were noted separately or in combination (Table 1; Fig. 1).

**Fig. 2:**
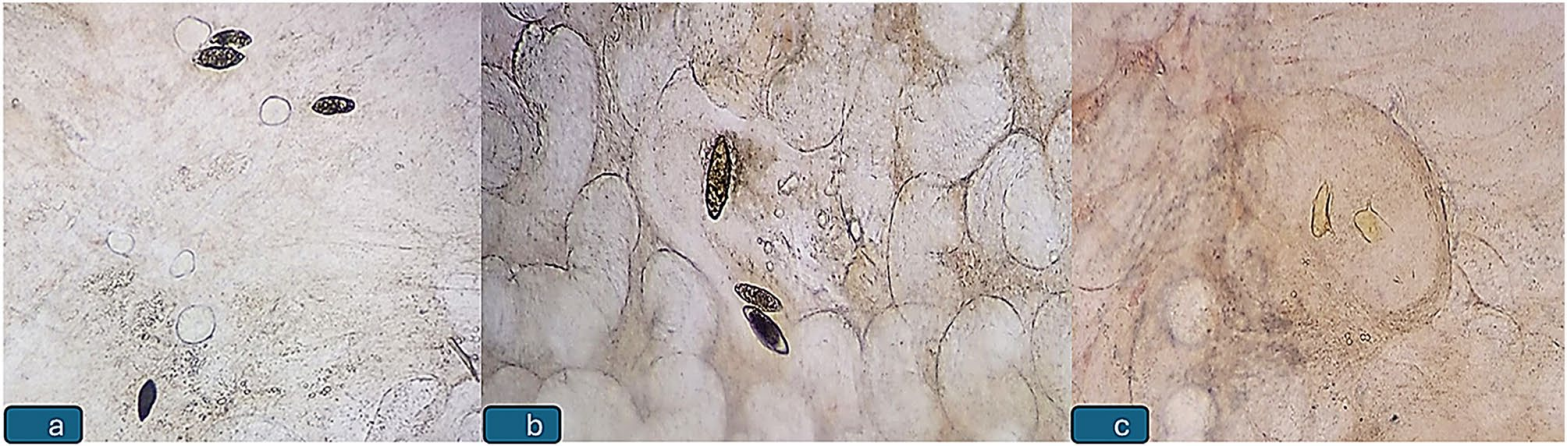
Non stained microscopic examination of Schistosoma mansoni calcified dead ova (**a**, **b**) and living ova (**c**) by crush biopsy test (× 100)

**Fig. 3:**
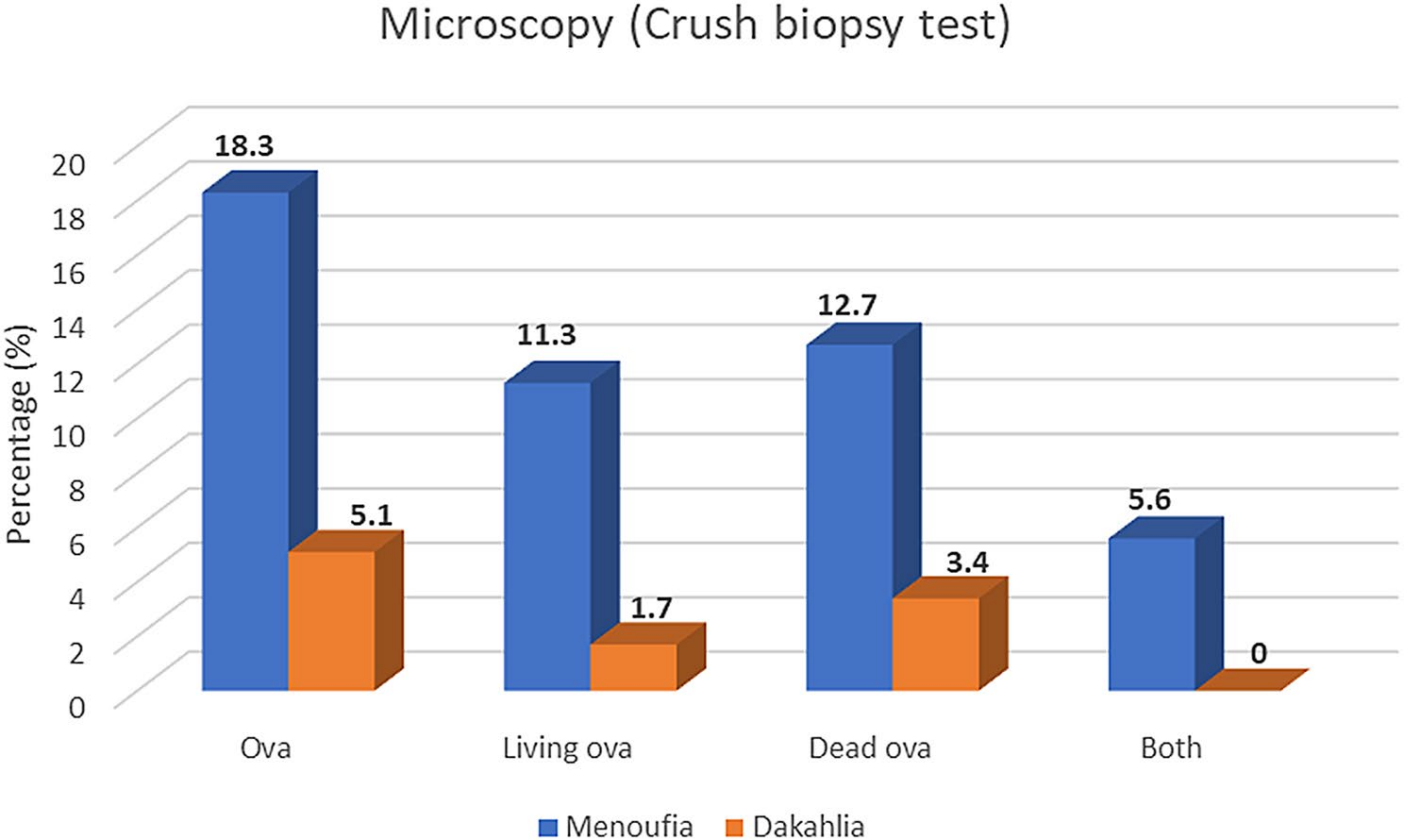
Comparison between Menoufia and Dakahlia governorates regarding the presence of schistosomal ova (living, dead or both) by crush biopsy test

A comparative analysis between the Menoufia and Dakahlia cohorts revealed significant differences in microscopic findings (direct examination of rectal biopsies using the crush technique) (*p* = 0.02). In Menoufia, 13 patients (18.3%) tested positive (12.7% dead ova, 11.3% living ova, and 5.6% both living and dead), whereas only three patients from Dakahlia showed positivity (3.4% dead ova, 1.7% viable ova). None from Dakahlia exhibited both forms (Table 2; Figs. 2 and 3). Residence patterns differed significantly between the two governorates (*p* = 0.001), with 35.2% of Menoufia participants residing in urban areas compared to only 6.8% in Dakahlia. Symptom profiles also varied: abdominal pain was predominant in Menoufia (50.7%), followed by diarrhea (32.4%), whereas Dakahlia participants primarily presented for follow-up of inflammatory bowel disease (IBD), altered bowel habits, or rectal cancer (55.9%) and only 13.6% of Dakahlia patients reported abdominal pain. Ultrasonography showed splenomegaly in 16.9% of Dakahlia patients and 1.4% in Menoufia (*p* = 0.001) (Table 3).

The relationship between symptoms and colonoscopy findings among the studied cases indicated no significant differences, except between diarrhea and polyps, as none of the 32 patients with diarrhea presented with polyps. The most common colonoscopic finding in patients with abdominal pain was polyps (4/41), whereas in those with rectal bleeding, it was erythema and congestion (4/22) (Table 3).

No correlation was found between schistosomiasis and IBD or colorectal cancer (CRC) (Table 4).

Ultrasound findings showed that periportal fibrosis was present in 22 cases (10.2%) across both groups, and the portal vein diameter remained normal in most instances, averaging 10.2 ± 7.35, as most patients did not report portal hypertension (results not shown in tables).

Regarding complete blood count (CBC) investigation, the hemoglobin level was 11.13 ± 2.52, and the platelet count averaged 245.8 ± 98.14 (data not shown).

**Table 1:** Comparison between positive and negative cases regarding patients’ characters

	Negative cases	Positive cases	Test of significance	P value
	(No ova)	N=16		
	N=114 (87.7%)	(12.30%)		
**Age / years Mean± SD (44.38±14.52)**				
<30	22(19.3)	1(6.3)	ꭓ^2^= 2.46	P= 0.293
30-60	76(66.7)	11(68.8)		
>60	16(14)	4(25)		
**Residence**				
Rural	92(80.7)	9(56.3)	ꭓ^2^= 4.84	P= 0.028*
Urban	22(19.3)	7(43.8)		
**Sex**				
Male	62(54.4)	13(81.3)	ꭓ^2^=4.15	P= 0.058*
Female	52(45.6)	3(18.7)		
**Indications for colonoscopy**				
Abdominal pain	40(35.1)	4(25)	ꭓ^2^=0.638	P=0.425
Bleeding per rectum	17(14.9)	5(31.3)	ꭓ^2^=2.66	P=0.103
Diarrhea	31(27.2)	1(6.3)	ꭓ^2^=3.32	P=0.069
Constipation	14(12.3)	2(12.5)	ꭓ^2^=0.001	P=0.980
Others (e.g. follow up for IBD, altered bowel habit, follow up for cancer rectum,…)	47(41.2)	4(25)	ꭓ^2^=1.55	P=0.213
**Ultrasound findings**				
**Organomegaly**	11(9.6)	4(25)	ꭓ^2^= 3.23	P= 0.072
**Splenomegaly**	10(8.8)	1(6.3)	ꭓ^2^= 0.193	P= 0.660
**Hepatosplenomegaly**	1(0.9)	3(18.8)	ꭓ^2^= 15.03	P= 0.006*
Colon Lesion				
Polyp	7(6.1)	6(37.5)	ꭓ^2^= 15.33	P= 0.001*
Ulcer	2(1.8)	6(37.5)	ꭓ^2^=31.04	P= 0.001*
Erythema with congestion	2(1.8)	10(62.5)	ꭓ^2^=61.79	P= 0.001*
Mass like	0	1(6.3)	ꭓ^2FET^=7.18	P= 0.123
Granular mucosa	0	4(25)	ꭓ^2FET^=29.41	P= 0.001*

Used test: Chi-Square, Fisher exact test, *statistically significant

**Table 2:** The relationship between patients, characteristics and the place of residence

	Total number	Menoufia	Dakahlia	Test of significance	P value
	N=130	N=71 (%)	N=59(%)		
**Age / years**					
<30	23(17.7)	10(14.1)	13(22.0)	ꭓ^2^=4.27	P=0.118
30-60	87(66.9)	53(74.6)	34(57.6)		
>60	20(15.4)	8(11.3)	12(20.3)		
**Residence**					
Rural	101(77.7)	46(64.8)	55(93.2)	ꭓ^2^=15.02	P=0.001*
Urban	29(22.3)	25(35.2)	4(6.8)		
**Sex**					
Male	75(57.7)	48(67.6)	27(45.8)	ꭓ^2^=6.29	P=0.012*
Female	55(42.3)	23(32.4)	32(54.2)		
**Complaint **					
Abdominal pain	44(33.8)	36(50.7)	8(13.6)	ꭓ^2^=19.86	P=0.001*
Bleeding per rectum	22(16.9)	18(25.4)	4(6.8)	ꭓ^2^=7.91	P=0.005*
Diarrhea	32(24.6)	23(32.4)	9(15.3)	ꭓ^2^=5.10	P=0.024*
Constipation	16(12.3)	8(11.3)	8(13.6)	ꭓ^2^=0.157	P=0.692
Others (e.g. follow up for IBD, altered bowel habit, follow up for cancer rectum,…)	51(39.2)	18(25.4)	33(55.9)	ꭓ^2^=12.64	P=0.0003*
History of Schistosoma Mansoni	0	0	0		
**Ultrasound findings**					
Organomegaly	15(11.5)	4(5.6)	11(18.6)	ꭓ^2^=5.34	P=0.021*
Splenomegaly	11(8.5)	1(1.4)	10(16.9)	ꭓ^2^=11.43	P=0.001*
Hepatosplenomegaly	4(3.1)	3(4.2)	1(1.7)	FET=0.692	P=0.626
**Colonoscopic lesions related to bilharziasis **					
Polyp	13(10)	7(9.9)	6(10.2)	ꭓ^2^=0.003	P=0.953
Ulcer	8(6.2)	7(9.9)	1(1.7)	ꭓ^2^=3.72	P=0.054*
Erythema with congestion	12(9.2)	12(16.9)	0	ꭓ^2^=10.98	P=0.001*
Mass like	1(0.8)	1(1.4)	0	FET=0.837	P=1.0
Granular mucosa	4(3.1)	4(5.6)	0	FET=3.42	P=0.126
**Microscopy Fig.** (3):					
Ova	16(12.3)	13(18.3)	3(5.1)	ꭓ^2^=5.22	P=0.02*
Living ova	9(6.9)	8(11.3)	1(1.7)	ꭓ^2^=4.58	P=0.032*
Dead ova	11(8.5)	9(12.7)	2(3.4)	ꭓ^2^=2.82	P=0.093
Both	4(3.1)	4(5.6)	0	FET=3.42	P=0.126

Used test: Chi-Square, Fisher exact test, *statistically significant

**Table 3:** Relation between symptoms and colonoscopy findings among studied cases

	Polyp *N* (%)	Ulcer *N* (%)	Erythema with inflammation *N* (%)	Mass like *N* (%)	Granular mucosa *N* (%)
Abdominal pain (*N* = 44)	4(9.1)	2(4.5)	2(4.5)	1(2.3)	2(4.5)
#	*P* = 0.805	*P* = 0.585	*P* = 0.336	*P* = 0.338	*P* = 0.604
Bleeding per rectum(*N* = 22)	3(13.6)	3(13.6)	4(18.2)	0	2(9.1)
#	*P* = 0.533	*P* = 0.133	*P* = 0.121	*P* = 1.0	*P* = 0.133
Diarrhea (*N* = 32)	0	1(3.1)	3(9.4)	0	0
#	*P* = 0.03*	*P* = 0.412	*P* = 0.974	*P* = 1.0	*P* = 0.572
Constipation (*N* = 16)	0	1(6.3)	1(6.3)	0	0
#	*P* = 0.154	*P* = 0.986	*P* = 0.66	*P* = 1.0	*P* = 1.0

Used test: Chi-Square, Fisher exact test, *statistically significant

**Table 4:** Correlation between schistosomiasis and Inflammatory bowel disease (IBD) and colorectal carcinoma (CRC)

		cancer	IBD
Dead ova			
	Pearson Correlation	− 0.092-	− 0.088-
	Sig. (2-tailed)	0.298	0.321
	N	130	130
Living ova			
	Pearson Correlation	− 0.087-	− 0.083-
	Sig. (2-tailed)	0.325	0.348
	N	130	130

## Discussion

Schistosomiasis has plagued the Egyptian population since ancient times. Efforts to eliminate the disease began in 1922 under the leadership of the Ministry of Health and Population (MOHP). Despite significant progress over the decades, complete eradication remains a persistent challenge [5].

This study investigated symptomatic patients with gastrointestinal complaints who attended the endoscopy units of the Departments of Hepatology and Gastroenterology at Mansoura University and the National Liver Institute, Menoufia University. These patients were residents of the Dakahlia and Menoufia governorates, located in the northeastern and southern portions of the Nile Delta—regions long recognized as endemic foci for *Schistosoma mansoni* [10, 12]. Both areas are predominantly agricultural, and a large proportion of their inhabitants engage in farming activities that involve frequent exposure to potentially contaminated irrigation water. Previous Egyptian [13–15] and international studies [16, 17] have consistently reported the persistence of schistosomiasis within such rural settings.

In this study, the percentage of colonic schistosomiasis among patients with colonic symptoms attending the endoscopy units (12.3%) was in agreement with Ahmed et al. [10], where active schistosomiasis was confirmed in 24 out of 193 patients from a rural community in the Egyptian Nile Delta referred for colonoscopy because of variable symptoms, with a prevalence rate of 12.4%.

The findings were inconsistent with those of Gad et al. [18], who reported a 20.83% prevalence of colorectal schistosomiasis among patients presenting with various gastrointestinal symptoms at the gastroenterology unit of Mansoura Specialized Medical Hospital from 2004 to 2009. This difference might be due to the longer study period, during which more patients were enrolled (984 versus 130). Also, Egypt was among the first nations to implement a comprehensive schistosomiasis control program. The program was carried out using different strategies, including mass treatment campaigns and health education, which over time resulted in a significant decline in the disease’s prevalence.

Regarding the higher infection rate in males than in females, these results were in agreement with those of Mones et al. and Ahmed et al. [5, 10], where more than 80% of positive cases were male. This can be explained by the fact that men were more likely to contract infections because, in our society, men are responsible for the family earnings most of the time; they are more employed in agricultural work and come in contact with water more frequently [10].

In this study, the infection rate was higher among rural inhabitants than among urban inhabitants. These results are consistent with the fact that schistosomiasis is mostly considered a rural disease. However, in recent decades, schistosomiasis has spread to peri-urban and urban areas. These data are believed to be explained by rural-urban migration in low- and middle-income nations and subsequent rapid and unplanned urbanization [19].

Clinical manifestations of intestinal schistosomiasis may initially be subtle; symptoms such as diarrhea, abdominal colic, and dysenteric features often go unnoticed, whereas chronic infection can progress to hepatosplenic involvement and portal hypertension [20, 21]. In this study, rectal bleeding and abdominal pain were the predominant symptoms prompting endoscopic evaluation. Notably, 25% of infected patients demonstrated organomegaly (hepatosplenomegaly in 18.8% and splenomegaly in 6.3%), implying either advanced or neglected disease [22]. These agreed with Ahmed et al. [10], who documented hepatomegaly and/or splenomegaly in 29.2% of positive cases. Similar rates have been reported in other Egyptian studies [23] and in Tanzanian populations [24], where hepatosplenomegaly percentages were 22.3%, 20.8%, 59.70%, and 13.73%, respectively.

In chronic active schistosomal colitis, patients may present with simultaneous acute and chronic inflammation within the same segment of the colon [25]. Advanced cases of intestinal schistosomiasis can lead to complications such as colonic or rectal polyps, narrowing of the intestinal lumen, or inflammatory masses that may mimic malignant tumors [26, 27].

In this study, positive cases showed morphological features consistent with both acute active infection (mucosal erythema and congestion), and features of chronicity (polyps, mucosal ulcerations, granular mucosa and mass like lesion). These findings were consistent with those reported by Ahmed et al. [10], who observed minor mucosal changes such as erythema and granularity in 33.4% of cases, while 66.6% presented with more severe lesions, including polyps, ulcers, and mass-like formations. However, a study by Gad et al. [18] involving 205 confirmed schistosomiasis cases, endoscopic evaluation revealed sigmoid colon sessile polyps in 2.42% of patients, mucosal congestion in 19%, petechiae in 5.4%, erosions or ulcers in 2.4%, and telangiectasia in another 2.4%. Notably, 67.8% of cases showed no visible mucosal abnormalities.

The relatively high proportion of schistosomal polyps in the present study (37.5%) was in line with both Ahmed et al. [10] and other Egyptian reports documenting higher polyp rates compared with non-African endemic areas such as Brazil [22]. In this study, small polyps were excised using cold snare polypectomy (CSP), while sessile lesions measuring 10–19 mm were removed using hot snare polypectomy (HSP), in accordance with the European Society of Gastrointestinal Endoscopy (ESGE) guidelines [28]. Given the substantial occurrence of schistosomal polyps, clinicians in endemic zones should consider schistosomiasis within the differential diagnosis of left-sided colonic lesions [29].

The choice to use the crush biopsy (squash) technique in the diagnosis of colonic schistosomiasis was guided by prior evidence of its diagnostic reliability. A large retrospective study from Saudi Arabia [30] demonstrated that among 2,458 colonoscopic cases, 216 patients (8.8%) had schistosomal colonic disease. Notably, ova were detected in fecal samples in only 11.1% of these patients, 49 individuals underwent crush biopsy, which revealed many *S. mansoni* eggs with a distinctive lateral spine. *Schistosoma mansoni* ova were not observed in the paraffin sections of 11 patients. The authors concluded that colonoscopy supported by crush biopsy was valuable for identifying characteristic lesions and confirming histological diagnosis [30].

A total of 25% of positive cases had organomegaly (either hepatosplenomegaly or splenomegaly alone). These results may be attributed to the considerable morbidity and mortality caused by chronic infections with *S. mansoni*, which lead to granuloma formation in the intestine and liver. Hepatic fibrosis may result in portal hypertension, which can subsequently lead to complications such as splenomegaly, esophageal varices, hematemesis, and mortality [31].

Schistosomal infection can resemble or coexist with inflammatory bowel disease (IBD) and colorectal cancer (CRC), warranting their inclusion in the present analysis. Misdiagnosis of schistosomal colitis as IBD is especially frequent in young adults, given the common involvement of the sigmoid colon and rectum. Clinicians should maintain a high index of suspicion for this condition, especially in patients presenting with nonspecific gastrointestinal symptoms and a history of travel to endemic regions [25]. Chronic schistosomal inflammation has also been linked to tumor development in organs such as the bladder, liver, and colon, primarily through sustained egg-induced granulomatous reactions, mucosal damage, microabscesses, and polypoid or neoplastic changes [32]. However, this study found no statistically significant association between schistosomiasis and either IBD or CRC.

This study has certain limitations, primarily its inclusion of symptomatic individuals only. The main objective was to identify schistosomiasis among patients presenting with gastrointestinal complaints and to highlight the continued presence of the disease in the region despite extensive national control programs. Additionally, several laboratory investigations—such as coagulation profiles, liver function tests, serological assays, and stool examinations—were not performed due to cost constraints and the practical challenges associated with bowel preparation before colonoscopy, which prevented proper stool sample collection on the day of the procedure.

Future research should incorporate these investigations to allow for a more comprehensive diagnostic assessment and to further support the utility of rectal mucosal examination using the crush (squash) biopsy technique. Comprehensive ultrasonographic evaluation, including measurements of splenic and portal venous diameters and assessment for collateral circulation, would also strengthen the diagnostic and clinical characterization of schistosomiasis in endemic areas.

## Conclusion

The crush (squash) biopsy technique proved to be a simple, sensitive, and dependable diagnostic tool for detecting colonic schistosomiasis, identifying both active and chronic infections. Despite ongoing national control efforts, schistosomiasis continues to pose a persistent public health challenge in the Egyptian Nile Delta, making elimination difficult. Sustained screening and surveillance remain essential, particularly among high-risk groups such as farmers and fishermen, who continue to play a central role in the transmission cycle due to their repeated exposure to infested water sources and the intermediate snail host.

## Data Availability

All data generated in the study are available with the corresponding author on reasonable demand.

## Abbreviations

IBD: Inflammatory bowel disease
MENA: Middle East and North Africa
MOHP: Ministry of Health and Population
WHO: World Health Organization
